# Supplementary figures and images for: In Silico Analysis of Glutamate Receptors in Capsicum chinense: Structure, Evolution, and Molecular Interactions
Source: Plants (Basel). 2024 Mar 12;13(6):812. doi: 10.3390/plants13060812 (PMC10975470; doi:10.3390/plants13060812)

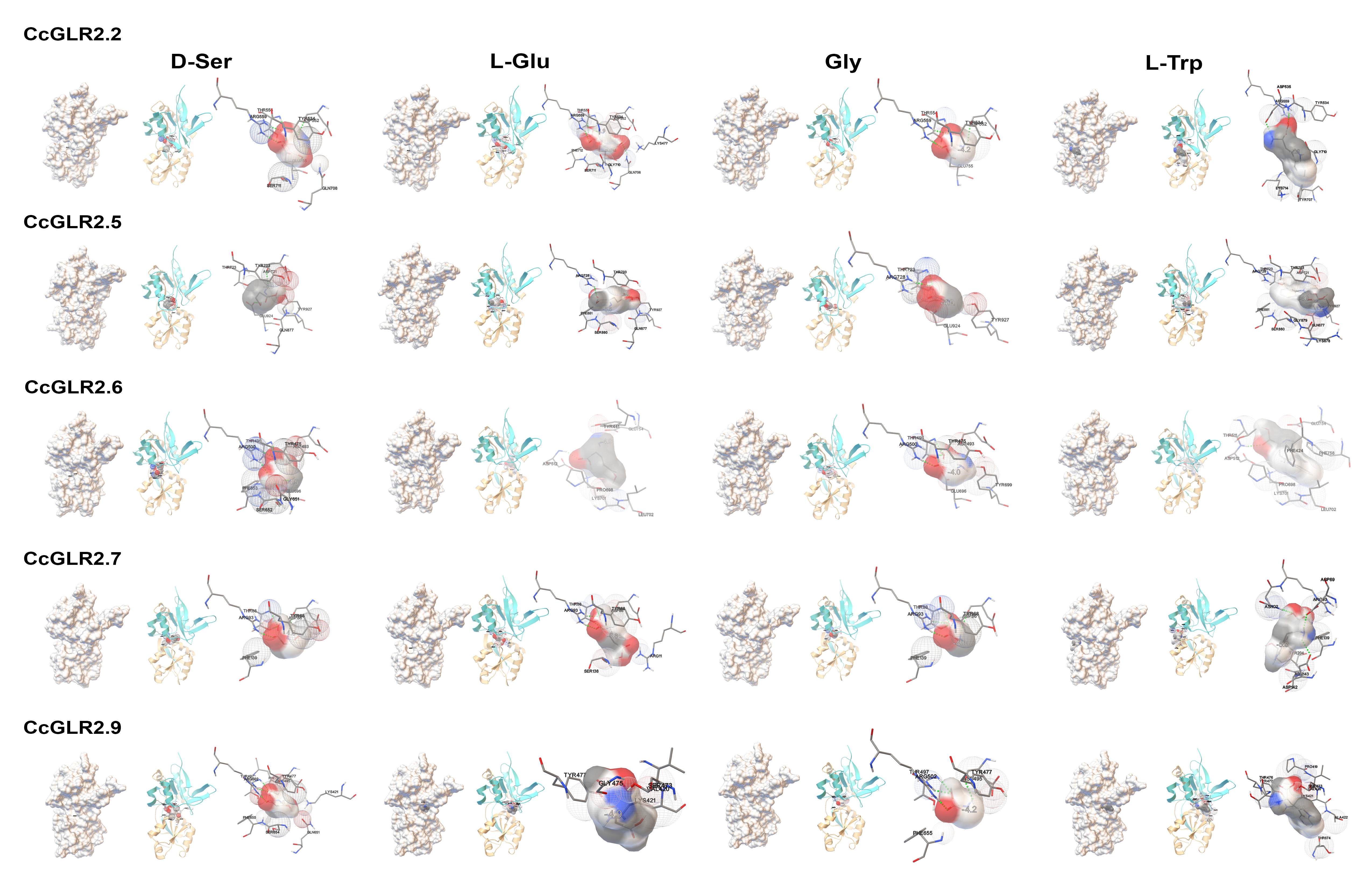

Supplement: Supplementary file 1 [file plants-13-00812-s001.zip › Figura S4_Fam_II.tif]

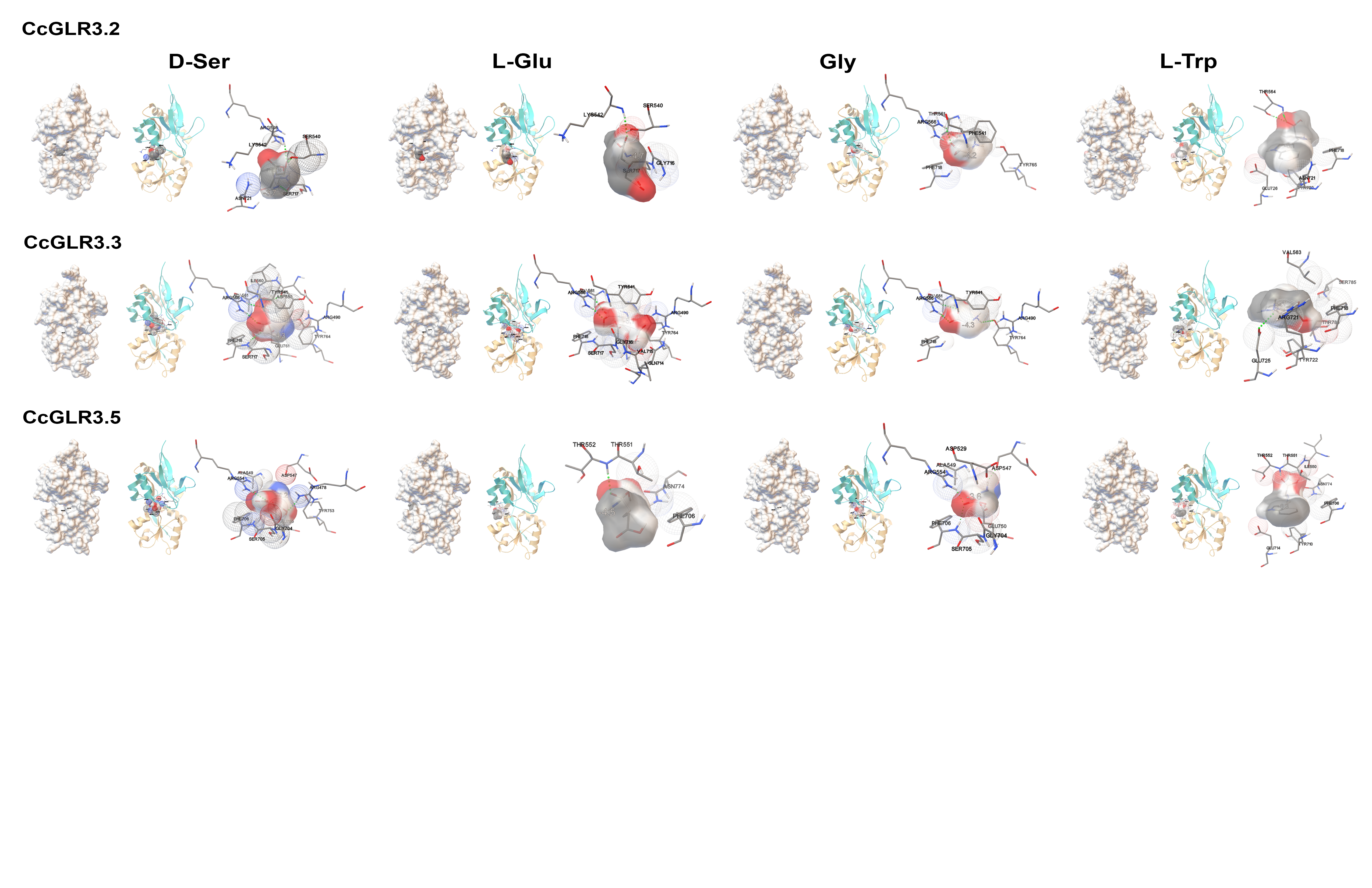

Supplement: Supplementary file 1 [file plants-13-00812-s001.zip › Figura S4_Fam_III.tif]

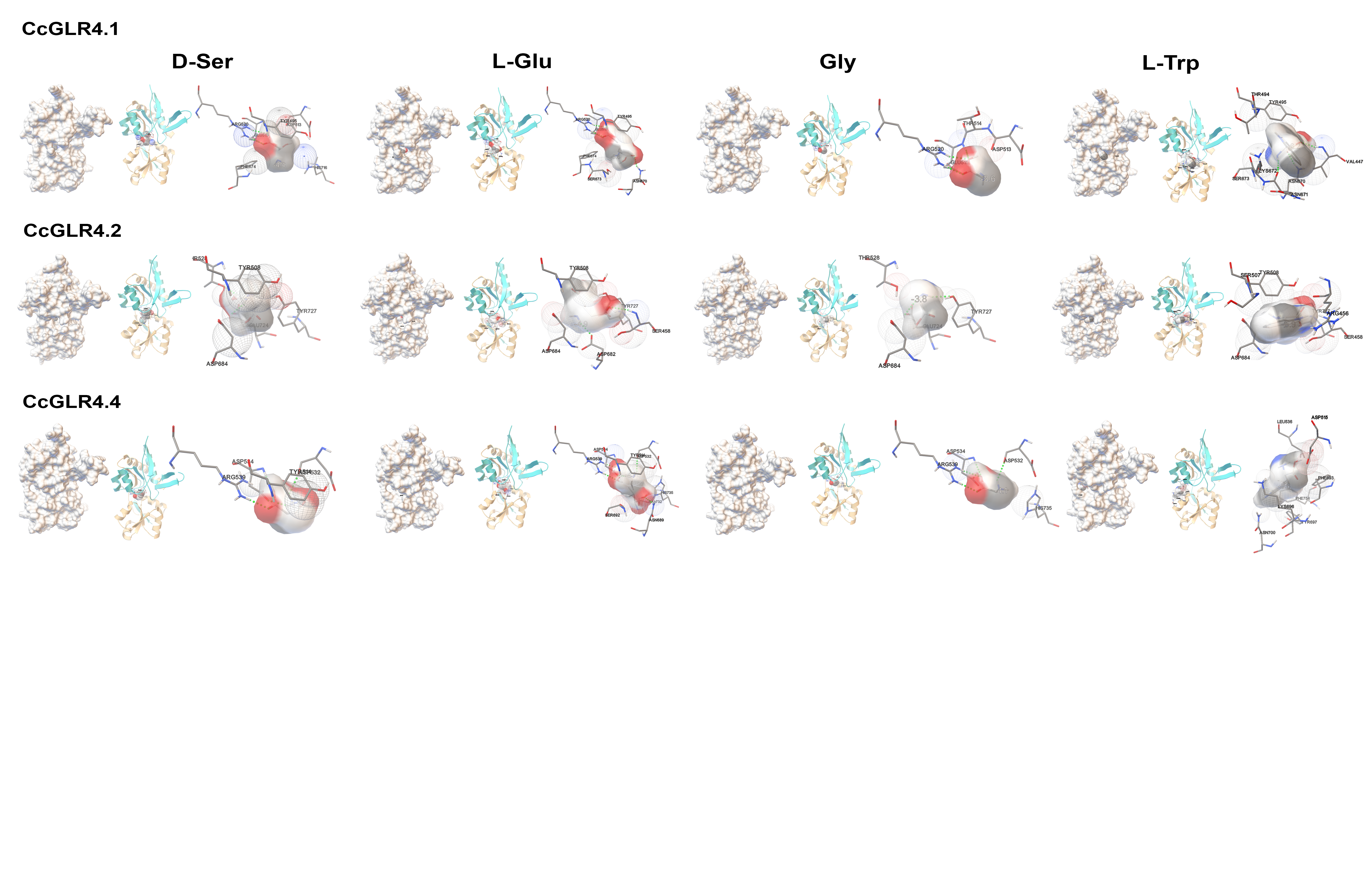

Supplement: Supplementary file 1 [file plants-13-00812-s001.zip › Figura S4_Fam_IV.tif]

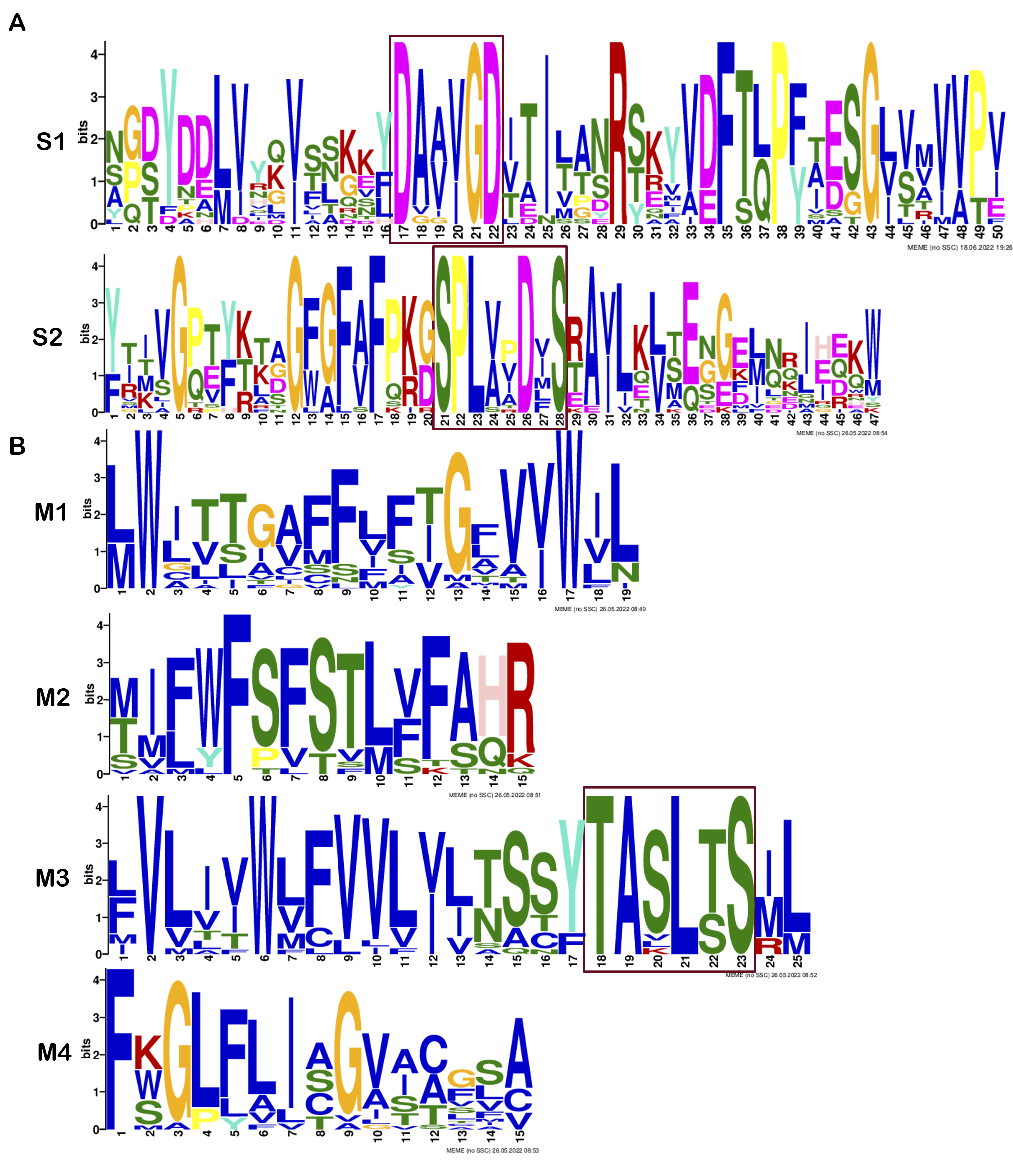

Supplement: Supplementary file 1 [file plants-13-00812-s001.zip › Figure S2.tif]

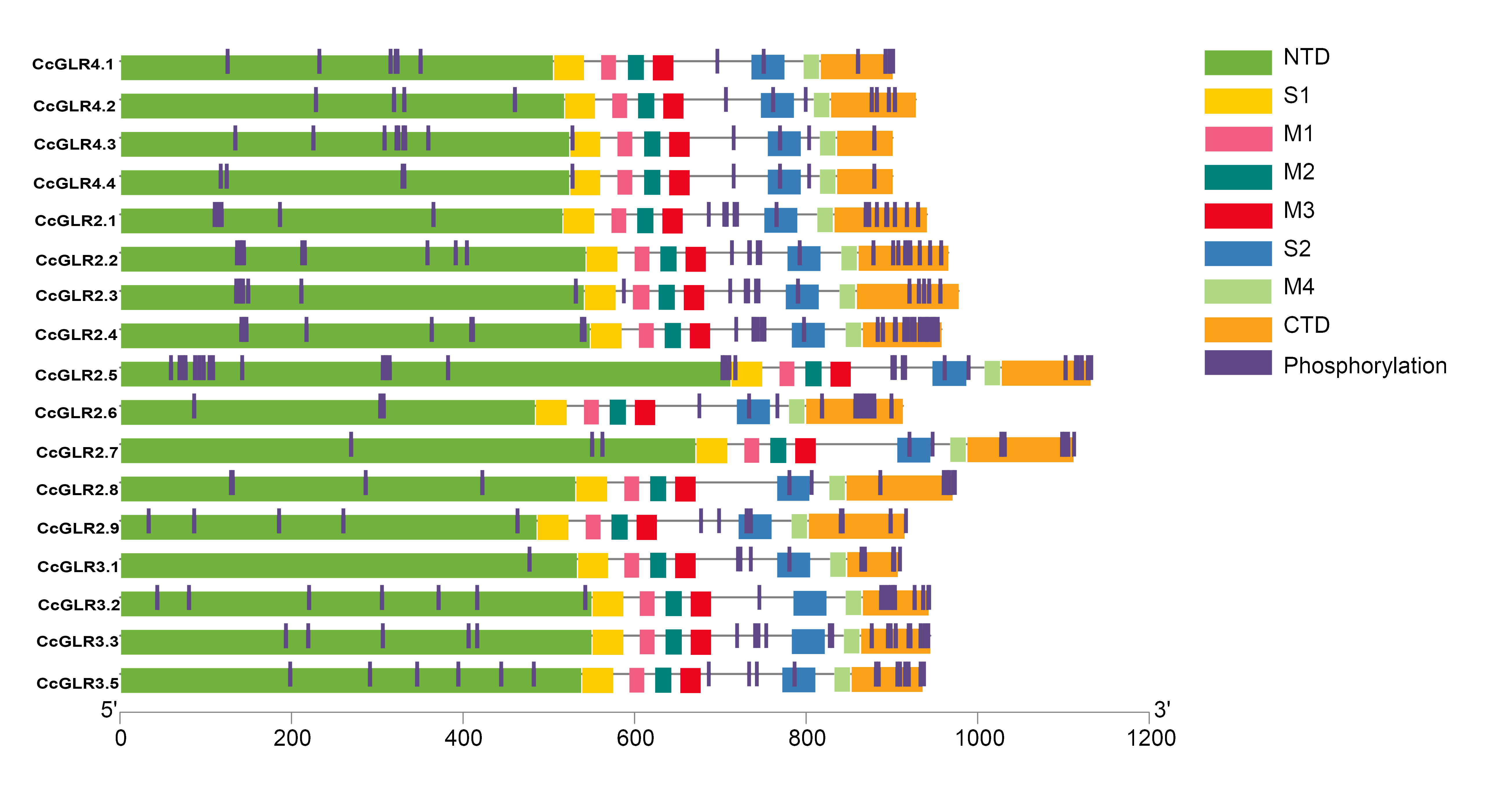

Supplement: Supplementary file 1 [file plants-13-00812-s001.zip › Figure S3.tif]

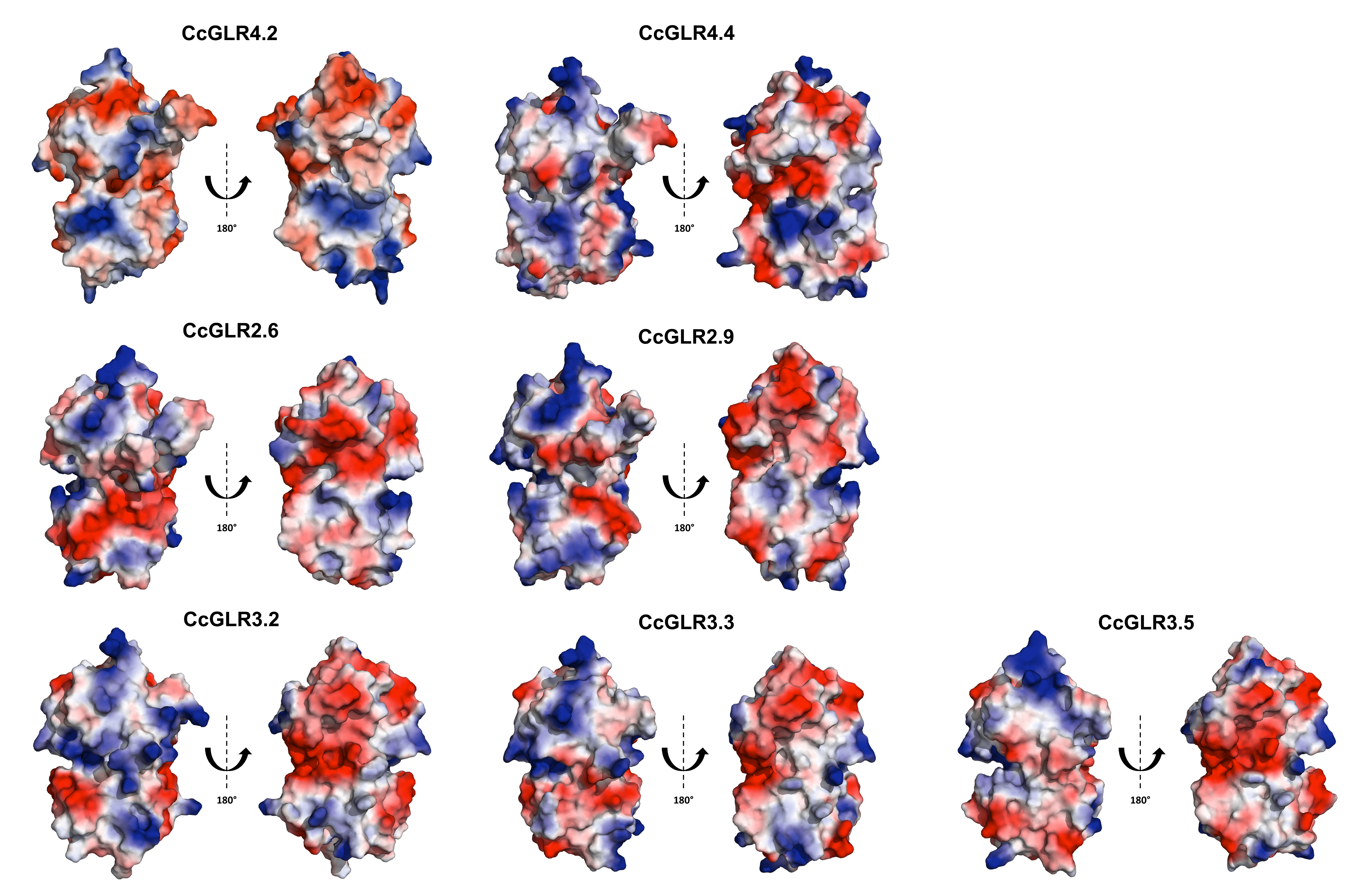

Supplement: Supplementary file 1 [file plants-13-00812-s001.zip › Figure S5.tif]
